# Deep learning–based prospective slice tracking for continuous catheter visualization during MRI‐guided cardiac catheterization

**DOI:** 10.1002/mrm.30574

**Published:** 2025-06-08

**Authors:** Alexander Paul Neofytou, Grzegorz Kowalik, Rohini Vidya Shankar, Karl Kunze, Tracy Moon, Nina Mellor, Radhouene Neji, Reza Razavi, Kuberan Pushparajah, Sébastien Roujol

**Affiliations:** ^1^ School of Biomedical Engineering and Imaging Sciences, Faculty of Life Sciences and Medicine King's College London London UK; ^2^ MR Research Collaborations Siemens Healthcare Limited Camberley UK; ^3^ Department of Paediatric Cardiology Evelina London Children's Hospital London UK

**Keywords:** cardiac catheterization, deep learning, MR guidance, passive tracking, real time

## Abstract

**Purpose:**

This proof‐of‐concept study introduces a novel, deep learning–based, parameter‐free, automatic slice‐tracking technique for continuous catheter tracking and visualization during MR‐guided cardiac catheterization.

**Methods:**

The proposed sequence includes *Calibration* and *Runtime* modes. Initially, *Calibration* mode identifies the catheter tip's three‐dimensional coordinates using a fixed stack of contiguous slices. A U‐Net architecture with a ResNet‐34 encoder is used to identify the catheter tip location. Once identified, the sequence then switches to *Runtime* mode, dynamically acquiring three contiguous slices automatically centered on the catheter tip. The catheter location is estimated from each *Runtime* stack using the same network and fed back to the sequence, enabling prospective slice tracking to keep the catheter in the central slice. If the catheter remains unidentified over several dynamics, the sequence reverts to *Calibration* mode. This artificial intelligence (AI)–based approach was evaluated prospectively in a three‐dimensional‐printed heart phantom and 3 patients undergoing MR‐guided cardiac catheterization. This technique was also compared retrospectively in 2 patients with a previous non‐AI automatic tracking method relying on operator‐defined parameters.

**Results:**

In the phantom study, the tracking framework achieved 100% accuracy/sensitivity/specificity in both modes. Across all patients, the average accuracy/sensitivity/specificity were 100 ± 0/100 ± 0/100 ± 0% (*Calibration*) and 98.4 ± 0.8/94.1 ± 2.9/100.0 ± 0.0% (*Runtime*). The parametric, non‐AI technique and the proposed parameter‐free AI‐based framework yielded identical accuracy (100%) in *Calibration* mode and similar accuracy range in *Runtime* mode (Patients 1 and 2: 100%–97%, and 100%–98%, respectively).

**Conclusion:**

An AI‐based prospective slice‐tracking framework was developed for real‐time, parameter‐free, operator‐independent, automatic tracking of gadolinium‐filled balloon catheters. Its feasibility was successfully demonstrated in patients undergoing MRI‐guided cardiac catheterization.

## 1 INTRODUCTION

Cardiac catheterization is a medical procedure commonly used for diagnosing and treating various heart conditions, particularly in patients with congenital heart disease.^1^ Such procedures are typically performed under fluoroscopic guidance, which has limitations, including poor soft tissue contrast and risks from ionizing radiation, especially for younger patients and those requiring multiple procedures.^2^, ^3^ Additionally, the medical team involved in such procedures risk orthopedic issues from heavy lead protective gear and direct radiation exposure.^4^

MRI is a promising alternative for guiding these procedures,^5^ already adopted clinically in several centers.^5^, ^6^, ^7^, ^8^, ^9^, ^10^, ^11^, ^12^, ^13^, ^14^, ^15^ MRI eliminates ionizing radiation risks and provides superior soft‐tissue‐contrast visualization and hemodynamic assessment via MR flow imaging.^6^, ^16^ MRI guidance typically uses dynamic single‐shot acquisition of one or more orthogonal slices. Balloon‐wedge catheters are used for navigation, with the balloon being filled with either gas or diluted gadolinium. The latter makes the catheter more conspicuous and generally allows for faster navigation.^7^ Visualization of gadolinium‐filled catheters can be enhanced using preparatory pulses, like nonselective saturation,^7^ black blood,^17^ or partial saturation (pSAT)^18^ pulses, with pSAT allowing simultaneous high‐contrast visualization of soft tissue, blood, and the catheter balloon.

However, the catheter frequently goes out of plane during navigation, which was observed for about 30% of the navigation time in a previous study.^13^ This requires manual repositioning of the imaging plane, which complicates and prolongs procedures. The T_1_ overlay approach reduces out‐of‐plane time^14^; however, it uses a larger slice thickness of 20 mm, which may reduce the value of image guidance in narrow anatomies.

A recently developed automated slice‐tracking technique improves catheter tracking during navigation.^19^ This approach uses real‐time image processing to identify the catheter coordinates. Despite its potential, it requires several patient and user‐specific parameters such as a bounding box definition encompassing the entire trajectory of the catheter as well as fine‐tuned parameters for signal thresholding, pattern matching, and temporal constraints. A robust, parameter‐free, and operator‐independent approach could streamline the workflow, support broader adoption, and enhance robustness across diverse patient populations. This study aimed to develop a novel parameter‐free, operator‐independent, artificial intelligence (AI)–based slice‐tracking technique. It was retrospectively compared with the original parameter dependent framework in 2 patients and prospectively evaluated in a three‐dimensional (3D)–printed heart phantom and 3 patients undergoing MR‐guided cardiac catheterization.

## 2 METHODS

### 2.1 Prospective AI‐based catheter‐tracking framework

**FIGURE 1:**
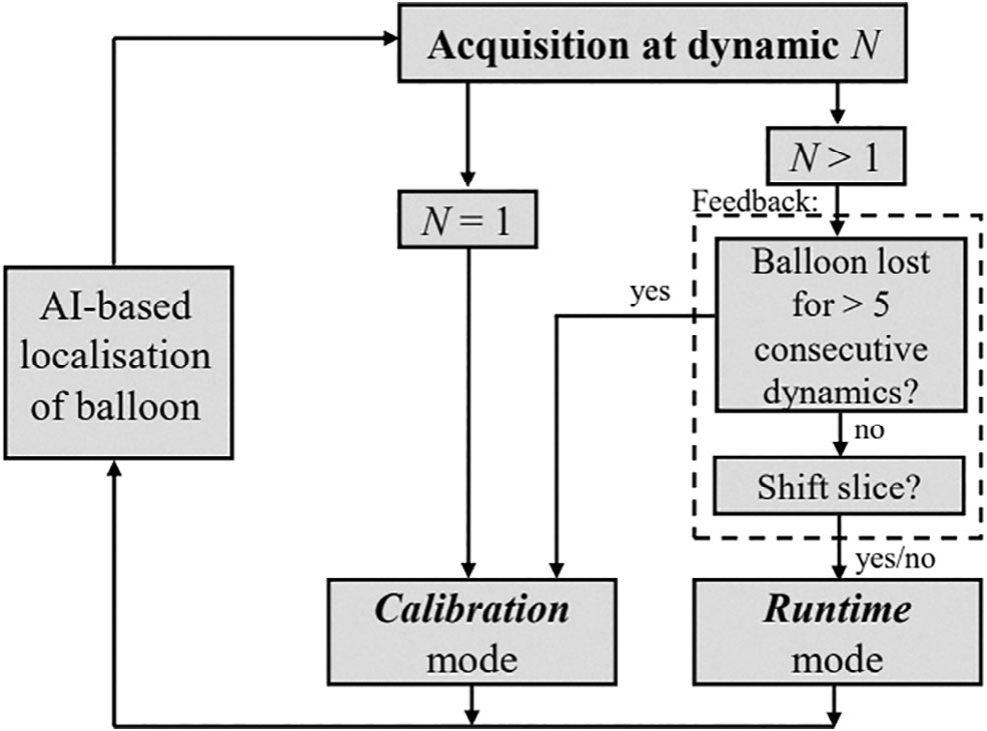
The prospective AI‐based slice tracking framework. The two imaging modes: *Calibration* and *Runtime* are depicted, where the *Calibration* acquisition is used to determine the initial coordinates of the balloon or redetermine its coordinates if lost. The *Runtime* stack aims to contain the balloon in the central slice of three contiguous slices and is re‐positioned if the balloon is detected in any outer slice by applying a slice shift towards the respective slice containing the catheter.

The prospective framework encompasses two imaging modes: *Calibration* and *Runtime* (refer to Figure 1), as previously described.^19^ In *Calibration* mode, a stack of fixed contiguous slices covering the entire space corresponding to the anticipated trajectory of the catheter is obtained to estimate the 3D coordinates of the catheter tip without prior knowledge of its location. Because these images are not intended to be used for visual guidance, a 90° nonselective saturation prepulse is applied before each image acquisition to maximize the contrast between the balloon and surrounding tissue and to facilitate automatic identification of the catheter balloon. Real‐time estimation of the catheter balloon 3D coordinates is then performed on the entire image stack using a deep learning–based approach described in the next section. If no catheter balloon is identified, the *Calibration* phase is repeated. Upon successful identification of the catheter balloon coordinates, the system transitions to *Runtime* mode, where three contiguous slices are automatically centered (i.e., the central slice) on the balloon's location and continuously acquired. The *Runtime* slice stack can be freely prescribed in any orientation. Because these images are intended to be used for image guidance, a pSAT prepulse with an angle of 50° is applied to provide simultaneous high‐contrast visualization of both the anatomy and the balloon.^18^ The same deep learning–based approach is then applied to extract the coordinates of the catheter balloon from each *Runtime* image stack, leading to three possible outcome cases: (1) catheter identified in the central slice; (2) catheter identified in one of the outer slices; or (3) catheter not identified.

In Cases 1 and 3, no adjustment is made to the subsequent *Runtime* slice stack. In Case 2, the subsequent *Runtime* stack is repositioned by applying a slice shift toward the slice containing the catheter, with a slice shift equal to the used slice thickness. If the catheter is not identified from multiple (i.e., *N* = 5) consecutive *Runtime* stacks, the sequence automatically reverts to the *Calibration* mode to re‐establish the initial coordinates before resuming in *Runtime* mode.

### 2.2 Deep learning–based identification of the catheter‐balloon 3D coordinates

The processing pipeline described here applies to both the *Calibration* and *Runtime* stack of slices. A deep learning model is used to segment the catheter balloon in each two‐dimensional (2D) image. This intermediate step is designated as “slice‐based segmentation.” This process is performed independently for each slice of a given stack (i.e., *Calibration* or *Runtime* stack). Occasionally, multiple regions may be identified within a single slice or across multiple slices in the stack—a scenario that arises when the catheter appears in adjacent slices or the model generates false positives. In such cases, the region with the highest signal intensity is selected. This final step, designated as “stack‐based segmentation,” concludes the segmentation process. The 3D coordinates of the catheter balloon are finally computed based on the center of mass of the selected region.

The deep learning model used in this postprocessing pipeline is a U‐Net with a ResNet‐34 encoder, which has been recently developed but only evaluated retrospectively and offline.^20^ In this study, this network has been implemented online, within the manufacturer's reconstruction environment, to enable its prospective use within the proposed tracking framework. This model was trained on semi‐artificial images, consisting of pSAT images acquired from 12 adult patients (480 images in total) undergoing standard diagnostic cardiac MRI examinations. An artificial catheter balloon signal was modeled as a 2D anisotropic Gaussian. The balloon was shaped by randomly selecting a standard deviation of 1.5 or 2.5 pixels in both the *x* and *y* directions and randomly rotating the shape between 0º and 360°. A randomly selected signal amplitude of 200%–400% of the underlying image signal intensity was also performed. The artificial balloon was then superimposed and centered at various relevant positions through the cardiovascular system in each image. This process generated a total of 4744 semi‐artificial images (3269 for training and 1475 for validation). All images were acquired in both transverse (20 slices) and coronal (20 slices) orientations, with the following imaging parameters: echo time/repetition time = 1.25/2.5 ms, flip angle = 70°, field of view (FOV) = 400 × 400 mm^2^, reconstructed/acquired resolution = 1.6 × 1.6/3.3 × 3.1 mm^2^, slice thickness = 10 mm, bandwidth = 1002 Hz/pixel, and GRAPPA factor = 2. The network was trained on coronal and transverse images and has been previously demonstrated to successfully detect the balloon in unseen training orientations (e.g., sagittal), demonstrating its generalization potential beyond the training data.^20^ More details of the network and semi‐artificial data generation can be found in Neofytou et al.^20^

### 2.3 Experimental evaluation

All imaging was performed on a 1.5T MRI scanner (MAGNETOM Aera; Siemens Healthineers, Erlangen, Germany) using a 32‐element spine array radiofrequency coil and an 18‐element body array coil during the acquisition. All experiments were conducted using a 4–6 French balloon wedge catheter (Arrow; Teleflex, Wayne, PA, USA). The balloon was filled with a 1% dilute concentration of Gadolinium (Dotarem; Guerbet, Villepint, France) for positive contrast visualization.

#### 2.3.1 Prospective evaluation in phantom

The proposed prototype framework was assessed in a 3D‐printed heart phantom. This phantom was submerged in a water‐filled plastic container, ensuring sufficient signal for visualizing the contrast between the shape of the 3D‐printed heart and the balloon of a wedge catheter. A 2D, single‐shot, balanced steady‐state free precession readout was used for both the *Calibration* and *Runtime* phases, with the following parameters: repetition time/echo time = 2/1 ms, pSAT angles of 90° for *Calibration* and 50° for *Runtime*, FOV = 300 × 300 mm^2^, matrix size = 128 × 128, slice thickness = 10 mm, temporal resolution = 205 ms, number of dynamics = 100, bandwidth = 1008 Hz/pixel, GRAPPA factor = 2, and stacks of nine coronal *Calibration* slices/three coronal *Runtime* slices. The catheter was navigated throughout the duration of the acquisition.

#### 2.3.2 Prospective patient study

Three female patients (age = 2.3 ± 1.4 years, weight = 9.8 ± 2.6 kg) with congenital heart disease (2 with atrioventricular septal defect, and 1 with ventricular septal defect and patent ductus arteriosus) were referred for MRI‐guided right heart catheterization and recruited for this study. This study was approved by our local institutional review board (REC reference: 16/LO/0173, IRAS project ID: 184807). The proposed prototype sequence was run in each subject during catheter navigation. All imaging parameters were matched to the phantom study apart from the following modifications to account for the varying children sizes: Patient 1 (FOV = 300 × 300 mm^2^, matrix size = 112 × 112, slice thickness = 7 mm, and dynamics = 59), Patient 2 (FOV = 400 × 280 mm^2^, matrix size = 160 × 160, slice thickness = 10 mm, dynamics = 117*), and Patient 3 (FOV = 350 × 350 mm^2^, matrix size = 160 × 160, slice thickness = 6 mm, and dynamics = 78*). *The number of dynamics here is the sum of multiple consecutive acquisitions. The *Calibration* (12 slices) and *Runtime* (3 slices) stacks were oriented in the coronal and sagittal planes, respectively.

#### 2.3.3 Retrospective patient study

The performance of image‐based catheter segmentation, using both the proposed and original parametric, operator‐dependent postprocessing approaches,^19^ was evaluated retrospectively in 2 male patients (age: 9.8 ± 2.6 years, weight = 26.6 ± 7.4 kg), with congenital heart disease (common arterial trunk and common arterial trunk), who underwent MRI‐guided right heart catheterization with data acquired using the original framework.

### 2.4 Data analysis

For both prospective studies, the postprocessing pipeline accuracy, sensitivity, and specificity in identifying the catheter balloon were determined for both the intermediate step (i.e., the slice‐based segmentation) and the complete process (i.e., the stack‐based segmentation). Slice‐based ground‐truth segmentation of the catheter balloon was manually defined for all images. A stack‐based ground truth was also generated by retaining only the manually segmented ground‐truth region across the slice stack with the highest signal intensity. In both analyses, a correct detection (i.e., true positive) was defined as a single segmented region with its center of mass intersecting the ground‐truth region of the balloon signal. The influence of balloon/background contrast‐to‐noise ratio on the true positive, false negative and false positive detections was also evaluated for the slice‐based analysis (see Supporting Information for further details).

For the retrospective study, the accuracy of the stack‐based segmentation was compared between the original parametric technique and novel parameter‐free AI technique.

## 3 RESULTS

### 3.1 Prospective evaluation in phantom

**FIGURE 2:**
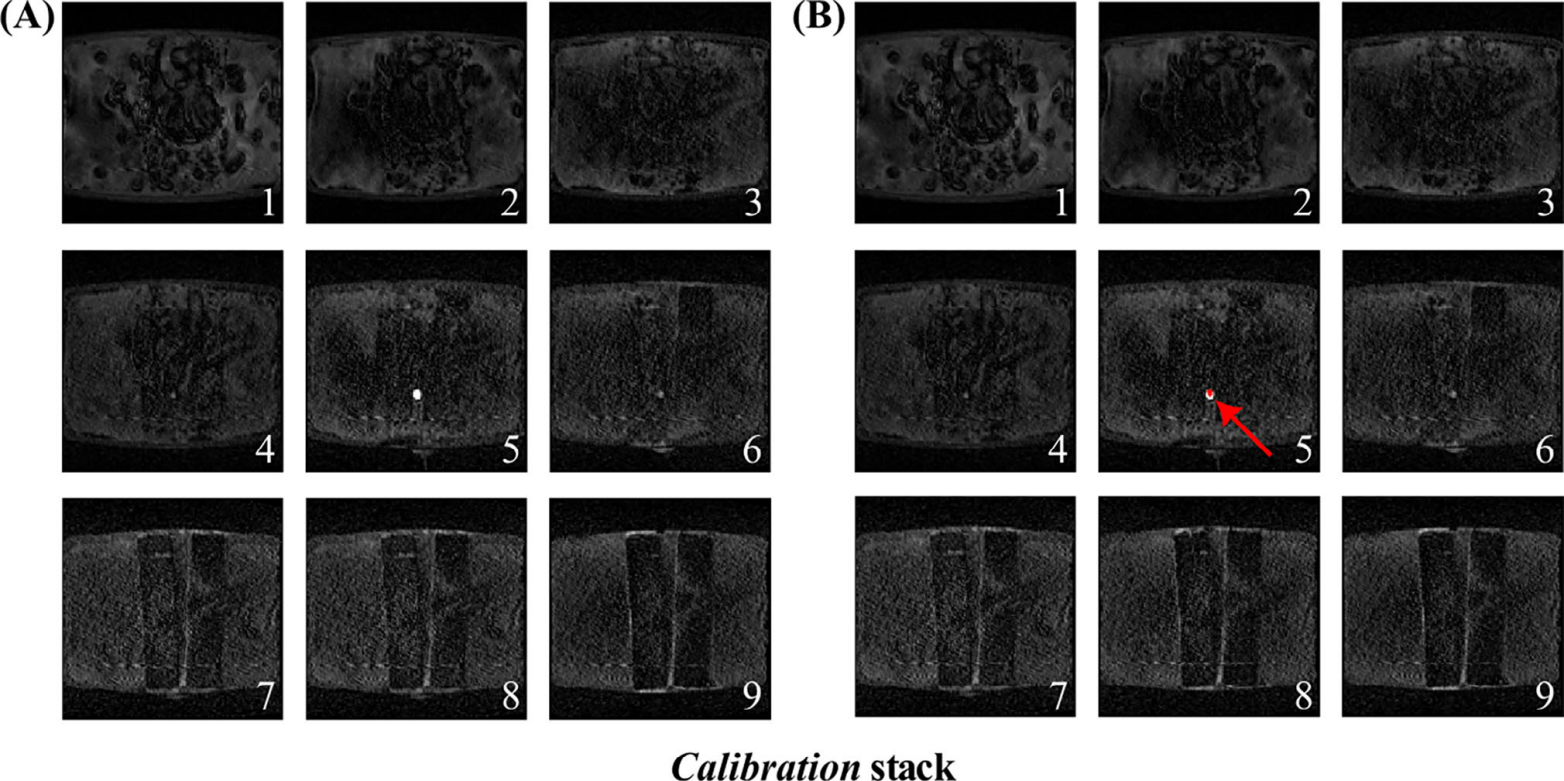
The Calibration stack acquired in dynamic 1 for the heart phantom. The stack is composed of 9 coronal slices used to determine the initial catheter coordinates before switching to Runtime mode. (A) The acquired magnitude images in the stack. (B) Overlay image of both magnitude images and AI‐generated masks, with the catheter detected in slice 5.

**FIGURE 3:**
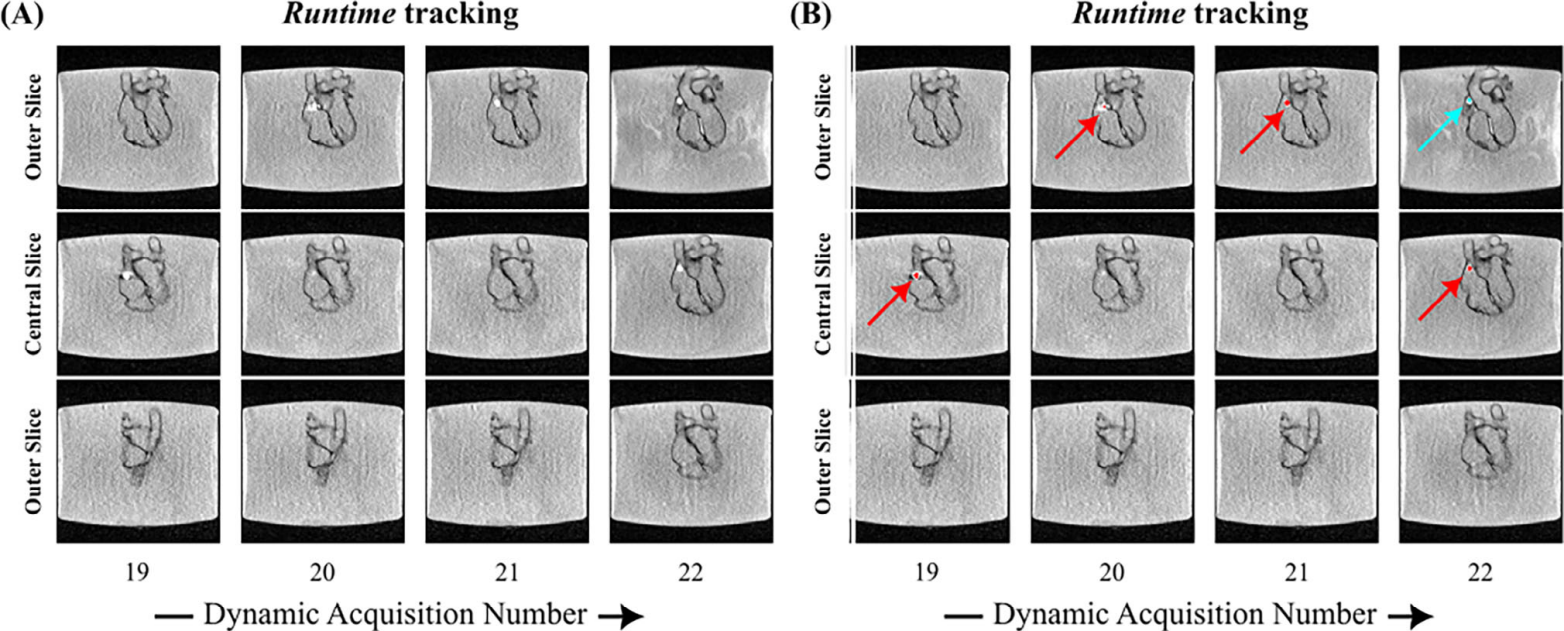
Temporal snapshots of the heart phantom depicting the recentering of the slices upon detection of the catheter in an outer slice: (A) Magnitude images, (B) Overlay of both magnitude images and AI‐generated masks. The red segmentation masks indicate the AI‐predicted segmentations with the highest intensity, while the cyan masks, initially identified in other slices (i.e., slice‐based segmentation) were discarded in the final post‐processing step (i.e., stack‐based segmentation).

Figure 2 depicts the *Calibration* stack (Dynamic 1), with the balloon automatically identified in Slice 5. In Figure 3, four dynamics illustrate the slice‐shifting process to center the slice with the detected catheter (see Video S1, showcasing all dynamics). The shift back to the central slice is observed, with a one‐dynamic delay due to latency.

Overall, the catheter was successfully tracked throughout the navigation, and *Runtime/Calibration* mode corresponded to 99%/1% of the imaging time. The accuracy, sensitivity, and specificity of slice‐based segmentation were all evaluated as 100% in *Calibration* mode and 96.6%, 94.5%, and 99.1%, respectively, in *Runtime* mode. More importantly, for the stack‐based segmentation analysis, the accuracy, sensitivity, and specificity all remained at 100% in the *Calibration* mode. In *Runtime* mode, all three metrics improved to 100%.

### 3.2 Prospective patient study

**FIGURE 4:**
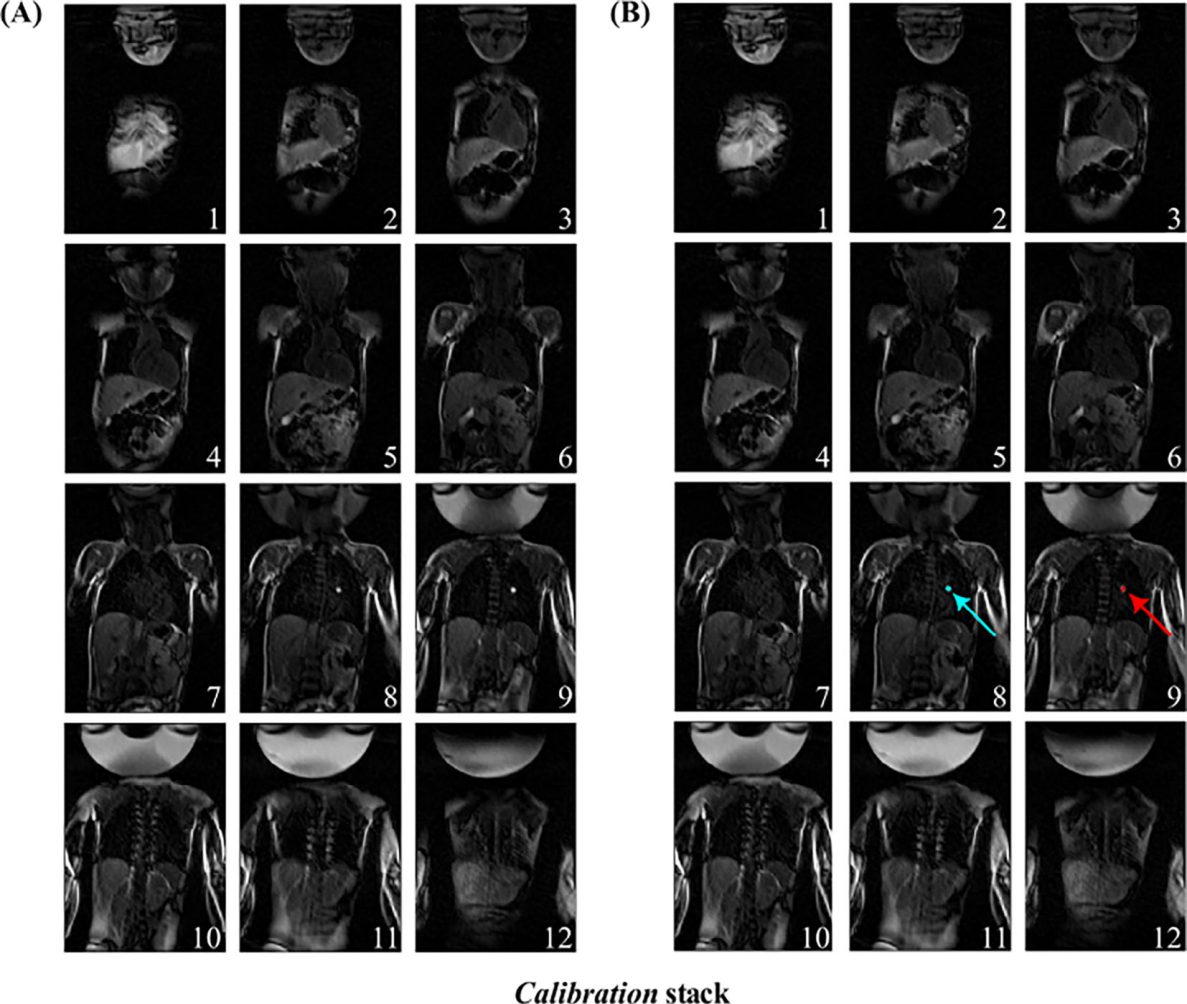
The *Calibration* stack acquired in dynamic 1 for patient 2. The stack is composed of 12 coronal slices through the patient to determine the initial catheter coordinates before switching to *Runtime* mode. (A) The acquired magnitude images in the stack. (B) Overlay image of both magnitude images and AI‐generated masks. The balloon is detected across two contiguous slices (i.e., 8 and 9), with slice 9 containing the highest intensity (red overlay) of the balloon signal and thus slice 9 is selected as the balloon's position.

**FIGURE 5:**
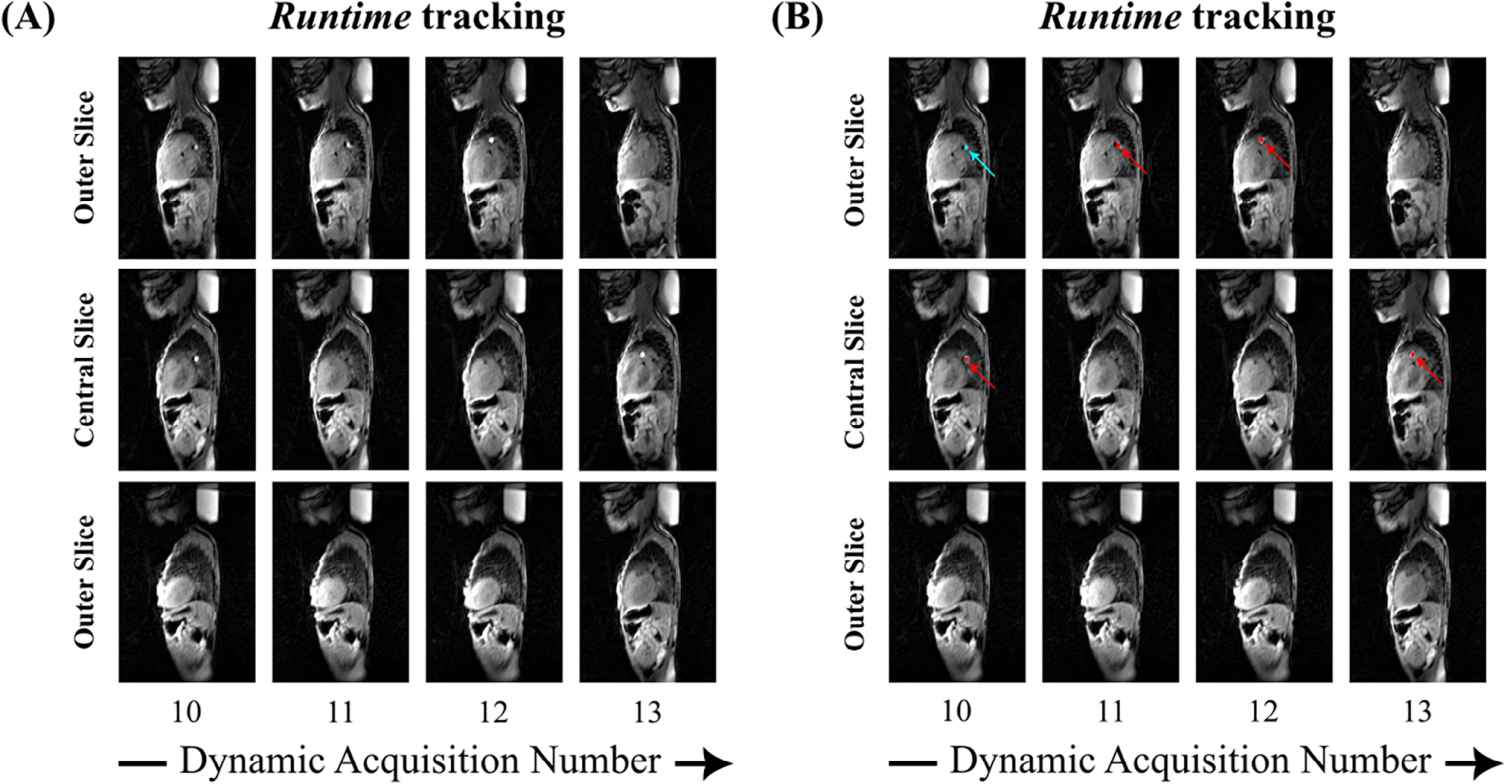
Temporal snapshots of patient 2 illustrating the recentering of the slices upon detection in an outer slice. (A) Magnitude images. (B) Overlay image of both magnitude images and AI‐generated masks. The red segmentation masks indicate the AI‐predicted segmentations with the highest intensity, while the cyan masks, initially identified in other slices (i.e., slice‐based segmentation) were discarded in the final post‐processing step (i.e., stack‐based segmentation).

Figure 4 displays the *Calibration* stack (magnitude images and AI‐predicted masks) for Patient 2, where the balloon was identified in Slices 8 and 9. The highest signal intensity was found in Slice 9, which was automatically selected as the balloon's slice position. Figure 5 illustrates five dynamics in *Runtime* mode from the same patient, highlighting the slice shifting to recenter the slices on the catheter. A delay of one dynamic was observed before the slice shifting occurred due to latency. Video S3 additionally shows correct instances for Patient 2, where the balloon is lost, necessitating a recalibration (e.g., at dynamic 79) of the balloon coordinates before returning to *Runtime* mode. Additional video examples for Patients 1 and 3 are provided in Videos S2 and S4, respectively. Video S2 provides an example of balloon manipulation and accurate detection and tracking of the balloon. Video S4 provides some examples of correct switching to *Calibration* mode (e.g., at Dynamic 16) after the catheter was lost.

On average, over all patients, the sequence was in *Runtime/Calibration* mode for 98%/2% of the time. Across all 3 patients, 100% of switches to *Calibration* mode were correct, when the balloon was lost. Overall, the catheter was successfully tracked throughout the navigation process in all patients.

#### 3.2.1 Slice‐based segmentation analysis

In *Calibration* mode, the accuracy/sensitivity/specificity (%) for each patient were 100.0/100.0/100.0 (Patient 1), 95.9/100.0/97.6 (Patient 2), and 100.0/85.7/100.0 (Patient 3), with an average of 98.6 ± 1.9/95.2 ± 6.7/99.2 ± 1.2. Similarly, in *Runtime* mode, the accuracy/sensitivity/specificity (%) for each patient were 93.7/91.4/96.3 (Patient 1), 94.1/92.7/95.1 (Patient 2), and 92.0/87.1/94.2 (Patient 3), with an average of 93.3 ± 0.9/90.4 ± 2.4/95.2 ± 0.9.

False‐negative/false‐positive detections were associated with lower balloon/background CNR than true‐positive detections for all patients: Patient 1 (10 ± 2/7 ± 2 vs. 19 ± 5; *p* < 0.0001 and *p* = 0.003), Patient 2 (16 ± 2/11 ± 2 vs. 23 ± 6; *p* < 0.0001 and *p* < 0.0001), and Patient 3 (11 ± 3/12 ± 4 vs. 13 ± 3; *p* = 0.04 and *p* = 0.28). However, this difference (false positive vs. true positive) was not statistically significant in Patient 3.

Some example cases (true positive and false negative) are shown in Figure S2.

#### 3.2.2 Stack‐based segmentation analysis

In *Calibration* mode, the accuracy/sensitivity/specificity were all 100% for all patients. In *Runtime* mode, the accuracy/sensitivity/specificity (%) for each patient were 99.4/98.2/100.0 (Patient 1), 97.6/92.1/100.0 (Patient 2), and 98.2/92.0/100.0 (Patient 3), with an average of 98.4 ± 0.8/94.1 ± 2.9/100.0 ± 0.0.

### 3.3 Retrospective patient study

On average, over all patients, the prospective sequence (i.e., original framework) was in *Runtime*/*Calibration* mode for 97%/3% of the time. Retrospective analysis of both the original and proposed methods yielded identical accuracy (100%) across all patients in *Calibration* mode. In *Runtime* mode, the accuracy for the 2 patients was 100% and 97% for the original method, and 100% and 98% for the proposed method, respectively.

### 3.4 Latency

The latency, measured as the reconstruction time for all three slices, postprocessing (i.e., AI inference), and feedback sent and received by the acquisition process, was about 1000 ms. Image reconstruction time was about 200 ms/slice. The computation time for postprocessing including AI inference was about 370 ms.

## 4 DISCUSSION

In this proof‐of‐concept study, we developed a parameter‐free, operator‐independent AI‐based slice‐tracking framework for continuous catheter visualization during navigation. Its feasibility was demonstrated prospectively in a 3D‐printed heart phantom and 3 patients undergoing MRI‐guided cardiac catheterization. This framework achieved high accuracy, sensitivity, and specificity for catheter detection.

The proposed AI‐based framework performed similarly to the original parametric, operator‐dependent method but without requiring patient‐ or processing‐specific parameters. In patients, the catheter was in *Runtime* mode for 97%–98% of the time in both the prospective (using the proposed approach) and retrospective (using the original framework) studies. In contrast, previous work showed that the catheter was not visible up to 30% of the time using standard single‐slice imaging.^13^ This approach may enhance the robustness of automatic catheter tracking, potentially shortening procedural times and reducing the complexity of image guidance. Further studies are needed to evaluate these potential outcomes.

The network was trained on semi‐artificial images from adult patients acquired with a pSAT angle = 20º–50° and applied to images in this study with different contrast (i.e., *Calibration* data with full saturation), anatomy (i.e., pediatric patients including one as young as 6 months old and phantom) and orientation (i.e., sagittal) unseen during training. These results suggest promising robustness across different image conditions. However, performance was reduced for lower catheter‐to‐background CNR. Expanding the training data set (e.g., including semi‐artificial images with lower balloon‐to‐background CNR) could improve generalizability.

The current latency of the framework is suboptimal for real‐time use. Because the scanner lacked a GPU, a CPU‐based AI inference was performed sequentially after acquiring three contiguous *Runtime* slices, contributing to the delay. Offline GPU‐based inference using our network was about ∼10 ms/image, demonstrating the potential for real‐time capability with modern scanners now including GPUs.

Temporal resolution is also affected by the acquisition of three slices in *Runtime* mode. A solution may be single‐slice acquisition, although this may increase switches to *Calibration* mode as previously observed.^19^ MRI acceleration schemes, such as SMS imaging,^21^ may offer a solution and allow for the acquisition of orthogonal orientations during *Runtime* mode, enhancing anatomical information and catheter detection.

Although this study focused on positive contrast visualization of gadolinium‐filled balloons, retraining the network for negative contrast visualization, such as for CO_2_‐filled balloons, is an interesting future direction. Additionally, extending this framework to other interventional devices for different medical procedures could enhance its clinical utility.

This study has some limitations. First, the use of only the sagittal (patients) and coronal (phantom) orientations during *Runtime* limits the assessment of performance in other orientations. Second, the sample size was small, although this was a proof‐of‐concept study aimed to demonstrate the feasibility of the proposed AI‐based tracking framework. These preliminary results warrant evaluation in a larger cohort to better assess the network's performance and its potential to facilitate and shorten cardiac catheterization procedures.

## 5 CONCLUSIONS

A framework using AI for prospective slice tracking was developed, enabling real‐time, parameter‐free, automatic tracking of gadolinium‐filled balloon catheters. The feasibility of this method was successfully demonstrated in patients undergoing MRI‐guided cardiac catheterization procedures.

## CONFLICT OF INTEREST

At the time of writing, APN held a PhD studentship partly funded by Siemens Healthcare Ltd., and KK is an employee of Siemens Healthcare Ltd.

## Supporting information


**Figure S1.** Histogram plots showing balloon‐to‐background contrast‐to‐noise ratio (CNR) values in Runtime mode for (*top to bottom*) true positives (TPs), false negatives (FNs), and false positives (FPs) in Patient 1 (A), Patient 2 (B), and Patient 3 (C). The mean CNR values (%) for TPs, FNs, and FPs were 19, 10, and 7 for Patient 1; 23, 16, and 11 for Patient 2; and 13, 11, and 12 for Patient 3, respectively. FN/FP detections were associated with lower balloon/background CNR than TP detections for all patients: Patient 1: 10 ± 2/7 ± 2 versus 19 ± 5 (*p* < 0.0001 and *p* = 0.003); Patient 2: 16 ± 2/11 ± 2 versus 23 ± 6 (*p* < 0.0001 and *p* < 0.0001); and Patient 3: 11 ± 3/12 ± 4 versus 13 ± 3 (*p* = 0.04 and *p* = 0.28). However, this difference (FP vs. TP) was not statistically significant in Patient 3. Some example cases (TP and FN) are shown in Figure S2.
**Figure S2**. (A) True positive (TP) examples. *Top*: Magnitude images only. *Bottom*: Magnitude images with predicted mask overlay. (B) False negative (FN) examples. A lower balloon‐to‐background contrast‐to‐noise ratio (CNR) is observed in FN cases compared with the TP cases.

**Video S1.** Dynamic depiction of the catheter detection in Calibration and Runtime modes for the heart phantom.

**Video S2.** Dynamic depiction of the catheter detection in Calibration and Runtime modes for Patient 1.

**Video S3.** Dynamic depiction of the catheter detection in Calibration and Runtime modes for Patient 2. Consecutive, separate acquisitions are combined in this video. Dynamics 1–39 correspond to the first acquisition, Dynamics 40–78 to the second acquisition, and Dynamics 79–117 to the third acquisition.

**Video S4.** Dynamic depiction of the catheter detection in Calibration and Runtime modes for Patient 3. Consecutive, separate acquisitions are combined in this video. Dynamics 1–39 correspond to the first acquisition, and Dynamics 40–78 to the second acquisition.
