# Supplementary material for: Deep learning–based prospective slice tracking for continuous catheter visualization during MRI‐guided cardiac catheterization
Source: Magn Reson Med. 2025 Jun 8;94(4):1626–34. doi: 10.1002/mrm.30574 (PMC12309870; doi:10.1002/mrm.30574)
Supplement: Supplementary file 1 — Figure S1. Histogram plots showing balloon‐to‐background contrast‐to‐noise ratio (CNR) values in Runtime mode for (top to bottom) true positives (TPs), false negatives (FNs), and false positives (FPs) in Patient 1 (A), Patient 2 (B), and Patient 3 (C). The mean CNR values (%) for TPs, FNs, and FPs were 19, 10, and 7 for Patient 1; 23, 16, and 11 for Patient 2; and 13, 11, and 12 for Patient 3, respectively. FN/FP detections were associated with lower balloon/background CNR than TP detections for all patients: Patient 1: 10 ± 2/7 ± 2 versus 19 ± 5 (p < 0.0001 and p = 0.003); Patient 2: 16 ± 2/11 ± 2 versus 23 ± 6 (p < 0.0001 and p < 0.0001); and Patient 3: 11 ± 3/12 ± 4 versus 13 ± 3 (p = 0.04 and p = 0.28). However, this difference (FP vs. TP) was not statistically significant in Patient 3. Some example cases (TP and FN) are shown in Figure S2. Figure S2. (A) True positive (TP) examples. Top: Magnitude images only. Bottom: Magnitude images with predicted mask overlay. (B) False negative (FN) examples. A lower balloon‐to‐background contrast‐to‐noise ratio (CNR) is observed in FN cases compared with the TP cases. [file MRM-94-1626-s002.docx]

**Supporting Information**

**Influence of balloon/background CNR on catheter detection**

The balloon/background CNR values were determined as follows. For the signal intensity calculations, the saved ground truth masks (for TP/FN vs CNR) and predicted masks (for FP vs CNR), which segmented the balloon signal were utilized. The mean signal intensity within a given mask was used to determine the signal of the balloon. To determine the mean signal intensity of the background tissue, the ground truth/predicted mask was dilated to about three times its original size. The mean pixel intensity value of background tissue was then determined in the region of the dilated mask, excluding the original mask region. The noise, $n$, was approximated from the *difference image* between two consecutive real-time images using the following formula:

$$n=\frac{SD}{\sqrt{2}} ,$$

where $SD$is the standard deviation of the signal within a region of interest (ROI). The *difference image* was carefully chosen to ensure that the two consecutive images were acquired at similar respiratory positions. One noise measurement was obtained per slice. For this analysis, the ROI was drawn in the area near the balloon signal area in the difference image, providing a local estimate of noise. Welch's t-test was performed to compare the means of CNR values between true positive and false negative/false positive cases for each patient.

**
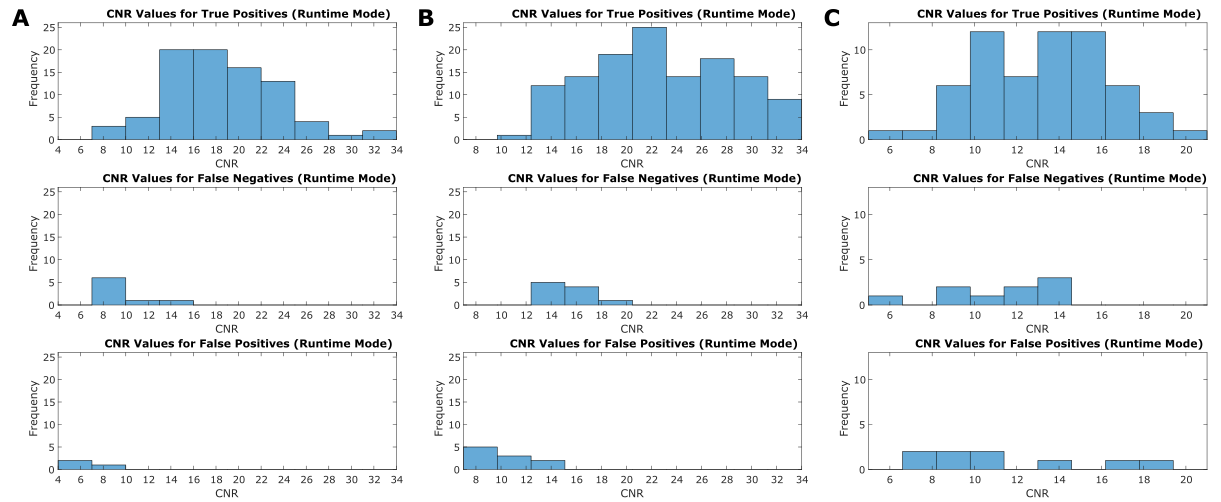
**

**Supporting Information Figure S1:** Histogram plots showing balloon-to-background contrast-to-noise ratio (CNR) values in Runtime mode for (top to bottom) true positives (TP), false negatives (FN) and false positives (FP) in patient #1 (A), patient #2 (B), and patient #3 (C). The mean CNR values (%) for TP, FN and FP were 19, 10 and 7 for patient #1, 23, 16 and 11 for patient 2, and 13, 11 and 12 for patient #3, respectively.

False negative/false positive detections were associated with lower balloon/background CNR than true positive detections for all patients: patient 1: 10±2/7±2 vs. 19±5, (p < 0.0001 and p = 0.003); patient 2: 16±2/11±2 vs. 23±6, (p < 0.0001 and p < 0.0001), and patient 3: 11±3/12±4 vs. 13±3, (p = 0.04 and p = 0.28), although this difference (FP vs TP) was not statistically significant in patient #3.

Some example cases (true positive and false negative) are shown in **Supporting Information Figure S2**.


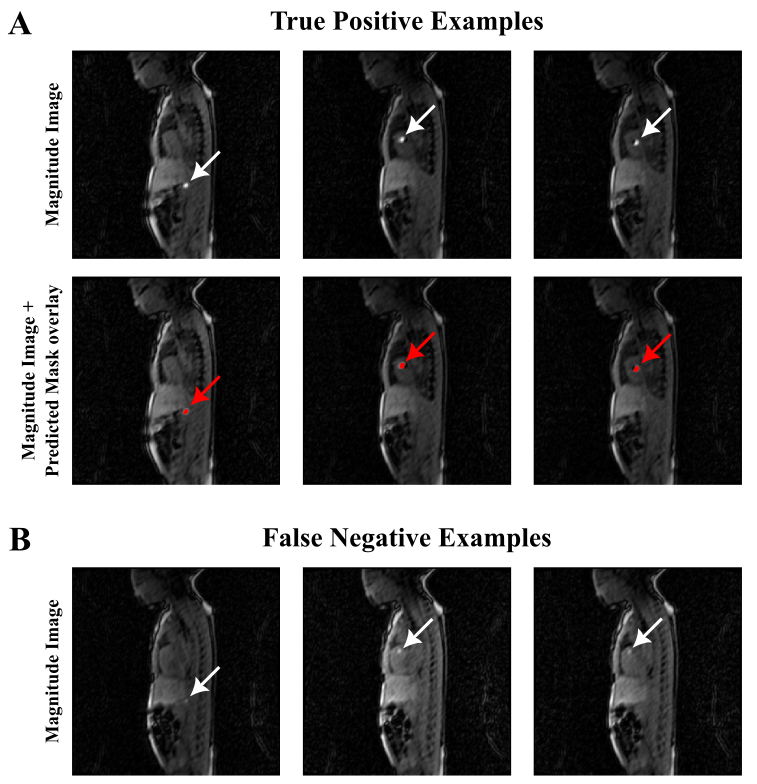


**Supporting Information Figure S2:** **(A)** True positive examples. (Top) Magnitude images only. (Bottom) Magnitude images with predicted mask overlay. **(B)** False negative examples. A lower balloon-to-background CNR is observed in false negative cases compared to the true positive cases.

**Supporting Information Video S1:** Dynamic depiction of the catheter detection in Calibration and Runtime modes for the heart phantom.

**Supporting Information Video S2:** Dynamic depiction of the catheter detection in Calibration and Runtime modes for patient 1.

**Supporting Information Video S3:** Dynamic depiction of the catheter detection in Calibration and Runtime modes for patient 2. Note: Consecutive, separate acquisitions are combined in this video. Dynamics 1–39 correspond to the first acquisition, dynamics 40–78 to the second acquisition and dynamics 79–117 to the third acquisition.

**Supporting Information Video S4:** Dynamic depiction of the catheter detection in Calibration and Runtime modes for patient 3. Note: Consecutive, separate acquisitions are combined in this video. Dynamics 1–39 correspond to the first acquisition and dynamics 40–78 to the second acquisition.
